# Pregnancy-related complications and perinatal outcomes following progesterone supplementation before 20 weeks of pregnancy in spontaneously achieved singleton pregnancies: a systematic review and meta-analysis

**DOI:** 10.1186/s12958-021-00846-6

**Published:** 2021-11-04

**Authors:** Hanglin Wu, Songying Zhang, Xiaona Lin, Jing He, Shasha Wang, Ping Zhou

**Affiliations:** 1 Department of Obstetrics and Gynaecology, Hangzhou Women’s Hospital, No. 369 Kun Peng Road, Hangzhou, 310008 Zhejiang China; 2 Assisted Reproduction Unit, Department of Obstetrics and Gynaecology, Sir Run Run Shaw Hospital, Zhejiang University School of Medicine, No. 3 Qingchun East Road, Hangzhou, 310016 Zhejiang China

**Keywords:** Low birth weight, Meta-analysis, Preeclampsia, Progesterone, Spontaneous conception

## Abstract

**Background:**

Progesterone supplementation is widely performed in women with threatened miscarriage or a history of recurrent miscarriage; however, the effects of early progesterone supplementation on pregnancy-related complications and perinatal outcomes in later gestational weeks remain unknown.

**Methods:**

Ovid MEDLINE, the Cochrane Library, Embase and ClinicalTrials.gov were searched until April 3rd, 2021. Randomized controlled trials regarding spontaneously achieved singleton pregnancies who were treated with progestogen before 20 weeks of pregnancy and were compared with those women in unexposed control groups were selected for inclusion. We performed pairwise meta-analyses with the random-effects model. The risk of bias was assessed according to the Cochrane Collaboration tool. The primary outcomes included preeclampsia (PE), and gestational diabetes mellitus (GDM), with the results presented as odds ratios (ORs) with 95% confidence intervals (CIs).

**Results:**

We identified nine eligible studies involving 6439 participants. The pooled OR of subsequent PE following early progestogen supplementation was 0.64 (95% CI 0.42–0.98, moderate quality of evidence). A lower OR for PE was observed in the progestogen group when the subgroup analysis was performed in the vaginal subgroup (OR 0.62, 95%CI 0.40–0.96). There was insufficient evidence of a difference in the rate of GDM between pregnant women with early progestogen supplementation and unexposed pregnant women (OR 1.02, 95% CI 0.79–1.32, low quality of evidence). The pooled OR of low birth weight (LBW) following oral dydrogesterone was 0.57 (95% CI 0.34–0.95, moderate quality of evidence). The results were affected by a single study and the total sample size of enrolled women did not reach the required information size.

**Conclusion:**

Use of vaginal micronized progesterone (Utrogestan) in spontaneously achieved singleton pregnancies with threatened miscarriage before 20 weeks of pregnancy may reduce the risk of PE in later gestational weeks. Among spontaneously achieved singleton pregnancies with threatened miscarriage or a history of recurrent miscarriage, use of oral dydrogesterone before 20 weeks of pregnancy may result in a lower risk of LBW in later gestational weeks. However, the available data were not sufficient to reach definitive conclusions, which highlighted the need for future studies.

**Supplementary Information:**

The online version contains supplementary material available at 10.1186/s12958-021-00846-6.

## Introduction

Progesterone, which is a female sex hormone, is secreted by the corpus luteum in the ovary to prepare the endometrium and to provide an adequate immune environment for the establishment of pregnancy [1]. Low progesterone levels have been associated with an increased risk of first trimester miscarriage [2, 3]. It has been postulated that progesterone supplementation in the early weeks of pregnancy may help to establish a sufficient immune response in early pregnancy and to prevent miscarriages.

The efficacy of progestogen therapy has been studied in women with threatened miscarriage and a history of recurrent miscarriage. Previous systematic reviews and meta-analyses have synthesized the literature and have suggested a potential benefit from progesterone therapy, especially for the subgroup of women with a history of three or more miscarriages [4–7]. The ACOG (American College of Obstetricians and Gynecologists) clinical management guidelines have concluded, “use of progestins for threatened early pregnancy loss is controversial, and conclusive evidence supporting their use is lacking. Women who have experienced at least three prior pregnancy losses, however, may benefit from progesterone therapy in the first trimester [8].”

Previous studies have focused on the live birth rate and miscarriage rate, and little attention has been given to the effects of early progesterone supplementation on pregnancy-related complications and perinatal outcomes in later gestational weeks. Two systematic reviews and meta-analyses both reported a significantly higher incidence of gestational diabetes mellitus (GDM) in women with singleton gestations receiving 17a-hydroxyprogesterone caproate for recurrent preterm birth prevention [9, 10]. The routes of administration and the types of progestogen for women with threatened early pregnancy loss are very different from those that are used to prevent preterm birth; therefore, whether the risk of GDM is higher in women who received early progesterone supplementation remains unknown. Tskhay V et al. found that dydrogesterone supplementation in the first and second periods of pregnancy significantly reduced the incidence of preeclampsia (PE) in women with higher-risk pregnancies; however, the study was retrospective and only women with risk factors for PE were included [11].

The aim of this systematic review and meta-analysis of randomized, controlled trials (RCTs) was to investigate the effects of early progesterone supplementation before 20 weeks of pregnancy on pregnancy-related complications and perinatal outcomes in later gestational weeks in spontaneously achieved singleton pregnancies.

## Methods

### Search strategy

This systematic review and meta-analysis was conducted and reported according to the PRISMA 2020 statement [12]. The study protocol was registered in PROSPERO (CRD42021245577). We searched Ovid MEDLINE, the Cochrane Library, Embase and ClinicalTrials.gov for RCTs published from the date of the inception of the specific database to April 3rd, 2021. No language limit was applied. Detailed search strategy can be found in Appendix S1. The reference lists of selected articles and reviews were hand searched to identify any relevant articles.

### Study selection

Titles and abstracts were initially screened to assess their potential eligibility, followed by a full-text examination to determine final eligibility. RCTs regarding women with singleton pregnancies who were treated with any type of progestogen for any reason before 20 weeks of pregnancy (compared with those women in unexposed control groups) were selected for inclusion. Studies concerning progesterone supplementation beyond the evaluation of outcomes were excluded. Previous studies have reported a higher risk of obstetric and perinatal complications in women achieving singleton pregnancy via assisted reproductive technology, compared with spontaneously achieved singleton pregnancies [13–15]. Therefore, only studies involving spontaneously achieved pregnancies were included. We excluded conference abstracts, crossover trials and quasi-RCTs. Two authors (HLW and SYZ) independently performed the selection process, with discrepancies being resolved by an additional reviewer (PZ).

### Outcome measures

We considered gestational hypertension, PE and GDM for our primary analyses. Gestational hypertension was defined as blood pressure ≥ 140/90 mmHg at least two occasions more than 4 h apart after 20 weeks’ gestation, PE was defined as gestational hypertension and the coexistence of one or both of the new-onset conditions, including proteinuria and other maternal organ dysfunction [16]. GDM was defined as any degrees of abnormal glucose metabolism during pregnancy that was not clearly overt diabetes prior to gestation [17]. The secondary interested pregnancy-related complications involved in this meta-analysis were placenta previa, placental abruption and postpartum haemorrhage. The perinatal outcomes included were low birth weight (LBW; < 2500 g), very low birth weight (VLBW; < 1500 g), preterm birth (PTB; < 37 weeks of gestation), small for gestational age (SGA; birth weight < 10th percentile), large for gestational age (LGA; birth weight > 90th percentile) and perinatal mortality (stillbirth and early or late neonatal death). In cases of certain discrepancies in the definition, we accepted the primary study authors’ definition, when relevant.

### Risk of bias assessment

Two independent reviewers (XNL and JH) undertook study quality assessment and data extraction. Any discrepancies were resolved by discussion within the review team. We assessed the risk of bias according to the Cochrane Collaboration tool for randomized trials [18]. Specifically, attention was focused on seven domains, i.e., random sequence generation, allocation concealment, blinding of participants and personnel, blinding of outcome assessment, incomplete outcome data, selective reporting and other biases. In each domain, bias was judged as high, low or unclear. The certainty of evidence was assessed using the Grading of Recommendations Assessment, Development and Evaluation criteria [19].

### Data extraction and synthesis

Relevant information from the included trials was extracted with a predefined data extraction sheet. The extracted data included study characteristics (published year, country and study sample size), patient characteristics (age and eligibility criteria), treatment information (randomization, type and duration of progestogen and treatment of control group) and outcomes. We obtained missing data and other relevant information via email requests to the authors of the studies.

Our primary analyses were based on the numbers of events and the numbers of ongoing pregnancies at approximately 20 weeks of gestation in each intervention and control group. For studies that did not report the denominators exactly as we defined them, we used the numbers of live births instead. We present the results as odds ratios (ORs) and associated 95% confidence intervals (CIs).

We performed pairwise meta-analyses with the random-effects model (the Mantel-Haenszel method) because we expected a great deal of heterogeneity in these studies, given that the studies were likely to incorporate different patient characteristics and use varying methodologies. We assessed statistical heterogeneity in each comparison with the I^2^ statistic and *p* value [20]. To calculate absolute effects, we utilized patient data to compare the risk differences for progestogen therapy versus placebo/no treatment.

Funnel plots were used for assessment of publication bias, and Egger’s test for asymmetry in funnel plot would only be performed if 10 or more studies were included [21]. Additional sensitivity analyses were performed by excluding studies with a high/unclear overall risk of bias. The potential impact of individual studies on the overall outcome of the analysis was investigated with a leave-one-out analysis; one study was sequentially omitted at a time to evaluate its effect in the outcome of the meta-analysis. Subgroup analyses of the primary outcomes were conducted, based on the type of administered progesterone and the eligibility criteria of the patients. Statistical analyses were conducted with Stata version 15.

We performed trial sequential analysis (TSA) to investigate the type I error in the aggregated result of meta-analyses. Repeated significance testing increases the risk of type I error in meta-analyses, and TSA can readjust the desired significance level by using the O’Brien-Flemming a-spending function. The risk for type I errors was set at 5%. The cumulative Z-curve of the meta-analysis was plotted to define sequential boundaries to assess type I and type II errors as well as the need for further trials in the field. During the analysis we also checked whether the total sample size of enrolled women reached the required information size that was needed to ensure adequate power. The TSA analysis was performed using the TSA version 0.9.5.10 Beta software (http://www.ctu.dk/tsa/).

## Results

### Study selection

**Fig. 1:**
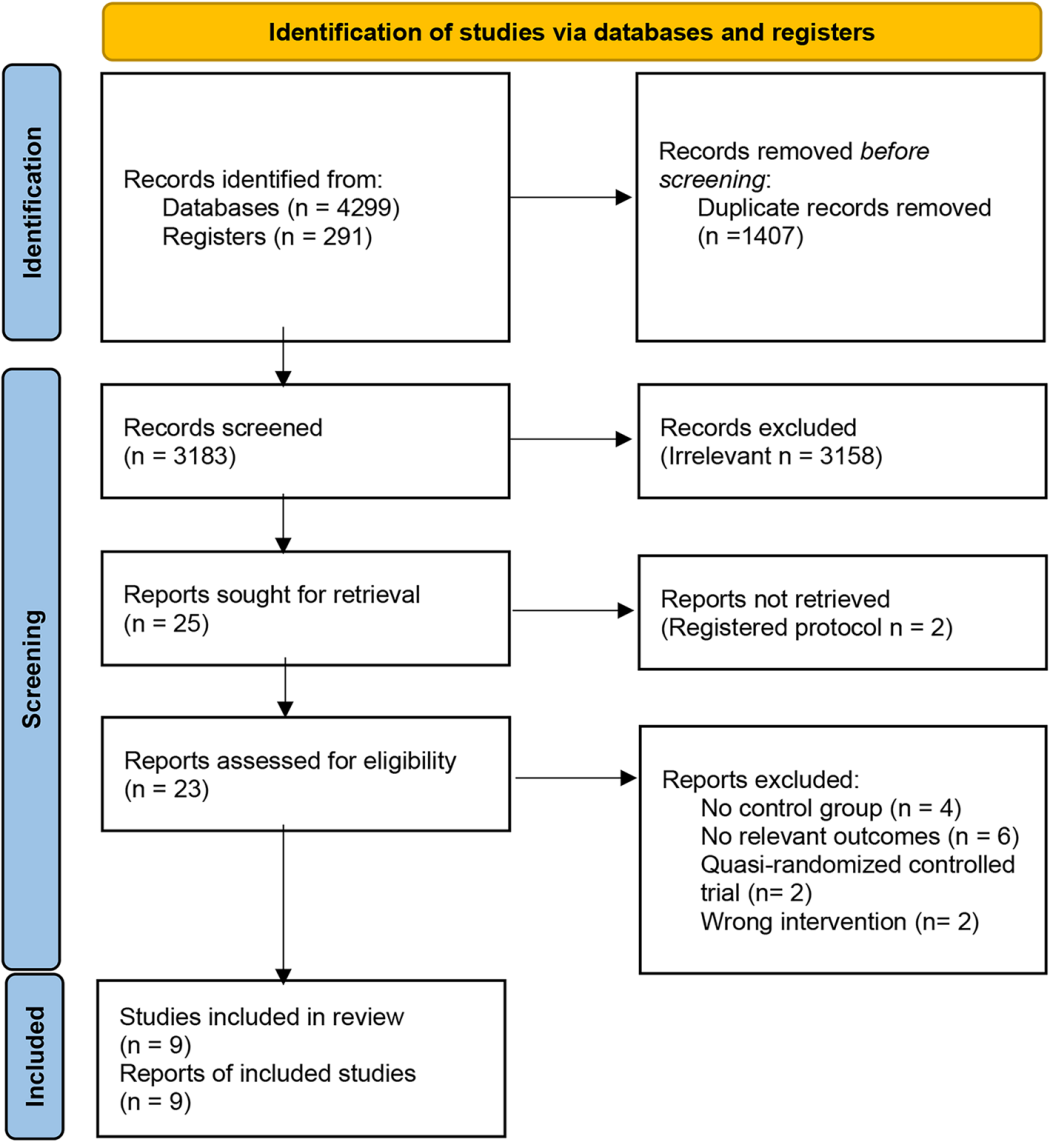
PRISMA 2020 flow diagram

Overall, 4590 citations were identified with the search and 23 potentially eligible articles were assessed for eligibility for the systematic review and meta-analysis. We excluded 10 of these references because there were no control groups [22–25] or relevant outcomes [26–31] in these studies, the authors were contacted, but there were no responses. Four studies were excluded due to a lack of an appropriate random process [32, 33] or progestogen therapy [34, 35]. The PRISMA flow diagram is shown in Fig. 1.

### Study characteristics

Table S1 presents the characteristics of the nine eligible trials involving 6439 participants [36–44] and the reasons for exclusion in study selection. Of the 6125 women who were included in the meta-analysis, 3055 women (49.9%) were randomized to the intervention group, and 3070 women (50.1%) were randomized to the control group. Eight of the trials were conducted in Asia or Europe. Additionally, seven studies enrolled patients with threatened abortions, whereas the other two studies included pregnant women with at least three previous unexplained miscarriages. Sample sizes varied from 60 to 4153. Seven studies used placebos as controls. When regarding the intervention, eight RCTs used oral or vaginal progesterone treatments, whereas the other RCT used rectal progesterone.

### Risk of bias and publication bias assessment of included studies

Figure S1 summarizes the findings from the risk of bias assessment for the included studies. Information concerning allocation concealment was obtained from Turgal M [41]. One of the included studies had a high risk of bias in “selective reporting” because the authors did not state the outcomes in the methods section, and information concerning the outcomes was obtained from Kumar A via email request [38]. Seven studies were double-blind, and only one study was classified as exhibiting an unclear risk for random sequence generation and allocation concealment [39]. Publication bias was assessed by using funnel plots (Fig. S2). The asymmetric nature of the funnel plots would suggest a possible publication bias. However, the assessment of publication bias in this review is particularly difficult, given that the number of studies was fewer than 10.

### Maternal outcomes

**Fig. 2:**
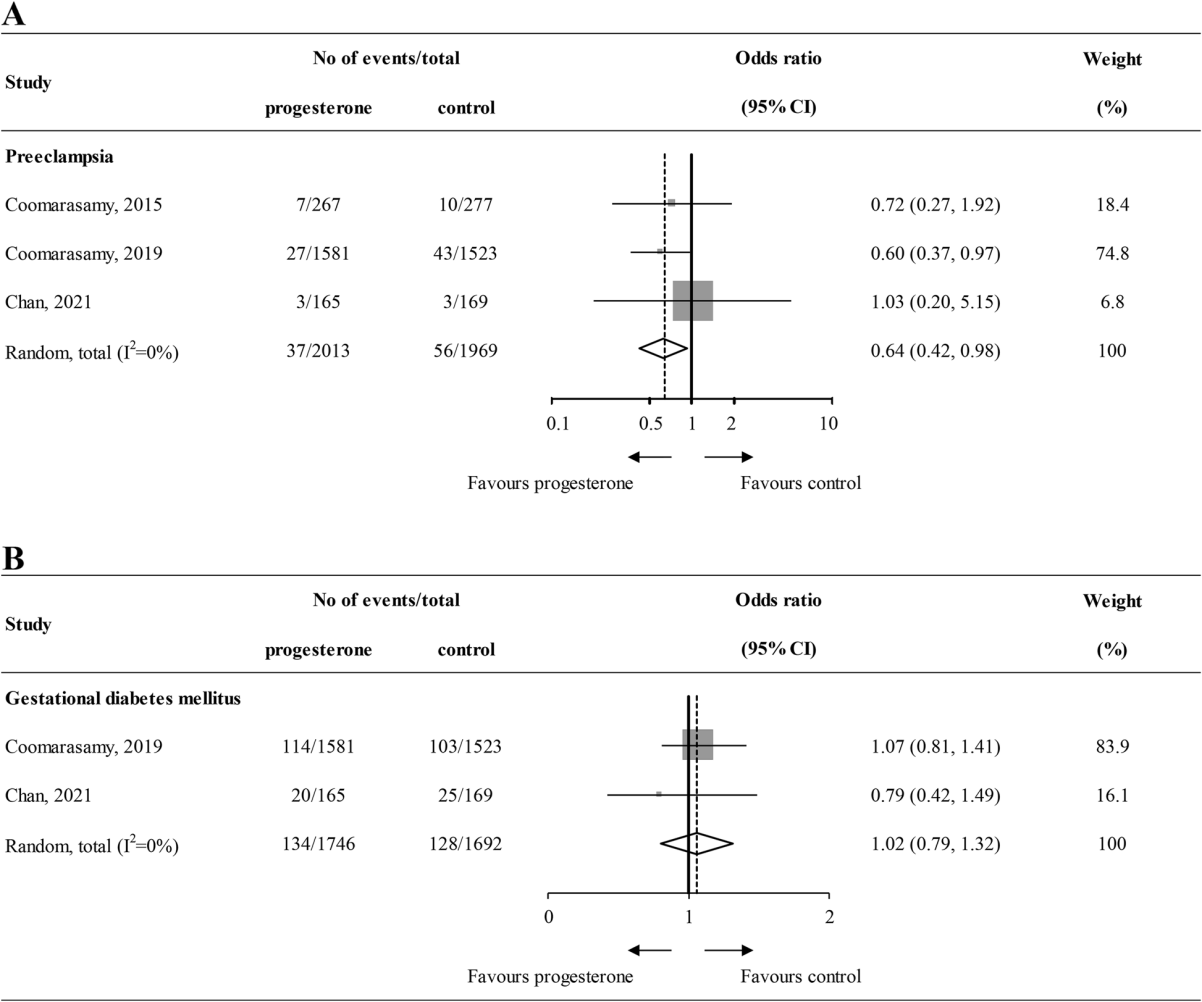
Forest plot diagrams of primary outcomes

Four studies reported the prevalence of pregnancy-induced hypertension. Three of them did not define the term “pregnancy-induced hypertension”, making us difficult to calculate the numbers of patients with gestational hypertension, therefore, meta-analysis was not performed. Three studies were involved in the meta-analysis of PE, including 2013 pregnancies in the progestogen groups and 1969 pregnancies in the control groups. The pairwise meta-analysis showed that there was a difference in the risk of PE when comparing pregnant women with early progestogen supplementation to unexposed pregnant women (OR 0.64, 95%CI 0.42–0.98, I^2^ = 0%, Fig. 2A). GDM was reported in two studies, the OR and the 95% CI of having GDM were 1.02 and 0.79–1.32, respectively, after progestogen supplementation, compared with no supplementation. There was no heterogeneity in the analysis (I^2^ = 0%, Fig. 2B). Additionally, we performed subgroup analyses for PE. A lower OR for PE was observed in the progestogen group when the subgroup analysis was performed in the vaginal subgroup (OR 0.62, 95%CI 0.40–0.96, I^2^ = 0%). However, in the other subgroup, no differences were shown between progestogen supplementation and no supplementation (Fig. S3).

There were three studies related to the complication of placenta previa. We did not find evidence of a difference in the risk of placenta previa among women after progestogen supplementation or no supplementation (OR 0.67, 95%CI 0.32–1.39, I^2^ = 0%, Fig. S4A). Two studies were involved in the meta-analysis of placental abruption, and no difference in the risk of placental abruption was found in women with progestogen supplementation compared with those women with no supplementation (OR 0.58, 95%CI 0.30–1.13, I^2^ = 0%, Fig. S4B). Moreover, postpartum haemorrhage was only reported in one study, and the postpartum haemorrhage rates in both groups were comparable (11% in the progestogen group and 12% in the control group) [42].

### Perinatal outcomes

Analysis of the data available from 4 RCTs showed that early progestogen supplementation in pregnant women resulted in a significant decrease in the LBW rate, compared with unexposed pregnant women (OR 0.57, 95%CI 0.34–0.95, I^2^ = 0%, Fig. S5A). in subgroup analyses, no differences were shown between progestogen supplementation and no supplementation (Fig. S6). Additionally, the risk of VLBW was not reported in the included studies. All the studies in our review reported PTB data, and the evidence was uncertain of a difference in the risk of PTB among women after either progestogen supplementation or no supplementation (OR 1.02, 95%CI 0.86–1.20, I^2^ = 0%, Fig. S5B). A sensitivity analysis was conducted on the outcome of PTB by excluding the study of Yassaee, and the summary estimate of the effect was not changed.

Five studies reported the prevalence of SGA. Progestogen supplementation in pregnant women was not associated with a lower rate of SGA than no supplementation (OR 0.86, 95%CI 0.58–1.27, I^2^ = 0%, Fig. S5C). Only one study reported the outcome of LGA, and the risk of LGA was 19% in both groups [42]. Seven of the nine included studies described the rate of PM. The OR of PM was 1.05 (95%CI: 0.58–1.90) after progestogen supplementation, compared with no supplementation. There was no heterogeneity in the analysis (I^2^ = 0%, Fig. S5D).

### Certainty assessment

Table 1 provides the certainty of evidence of the estimates for the outcomes. We downgraded evidence certainty to low or very low for most of the comparisons, mainly because of imprecision and the possibility of a publication bias. Moderately certain evidence showed that one additional woman was expected to be prevented from developing PE for every 125 pregnant women receiving progestogen, compared with those women in the unexposed control groups (number needed to treat: 125). The moderately certain evidence showed that one additional infant with LBW was expected to be prevented for every 34 pregnant women receiving progestogen, compared with those women in the unexposed control groups (number needed to treat: 34).

**Table 1:** Summary of meta-analysis estimates of effects, confidence intervals, and certainty of evidence

	Relative effect odds ratio (95% CI)*	Anticipated absolute effect, per 1000 patients† (95% CI)			No. of participants (studies)	Certainty of evidence	Number needed to treat (95% CI)
		Progestogen	Placebo/no treatment	Difference			
**Maternal outcome**							
Pre-eclampsia	**0.64 (0.42, 0.98)**	14	22	8 fewer (15 fewer to 1 fewer)	3982 (3 RCTs)	⊕ ⊕ ⊕○, moderate^4^	125 (67 to 1000)
Gestational diabetes mellitus	1.02 (0.79, 1.32)	61	58	3 more (12 fewer to 16 more)	3438 (2 RCTs)	⊕ ⊕ ○○, low^1, 4^	Not calculated (non-statistically significant)
Placenta previa	0.67 (0.32, 1.39)	6	8	2 fewer (7 fewer to 2 more)	3590 (3 RCTs)	⊕○○○, vey low^1, 2, 4^	Not calculated (non-statistically significant)
Placental abruption	0.58 (0.30, 1.13)	7	12	5 fewer (10 fewer to 1 more)	3219 (2 RCTs)	⊕○○○, very low^1, 2, 3, 4^	Not calculated (non-statistically significant)
**Perinatal outcome**							
Preterm birth	1.02 (0.86, 1.20)	113	107	4 more (14 fewer to 15 more)	4782 (9 RCTs)	⊕ ⊕ ○○, low^3, 4^	Not calculated (non-statistically significant)
Low birth weight	**0.57 (0.34, 0.95)**	52	81	29 fewer (53 fewer to 1 fewer)	935 (4 RCTs)	⊕ ⊕ ⊕○, moderate^4^	34 (19 to 1000)
Small for gestational age	0.86 (0.58, 1.27)	52	58	6 fewer (29 fewer to 9 more)	4145 (4 RCTs)	⊕○○○, very low^1, 3, 4^	Not calculated (non-statistically significant)
Perinatal mortality	1.05 (0.58, 1.90)	9	8	1 more (2 fewer to 5 more)	4632 (7 RCTs)	⊕○○○, very low^1, 2, 3, 4^	Not calculated (non-statistically significant)

*Significant results are in bold

†Data obtained directly from study sample (studies reporting outcome data)

^1^ Wide confidence intervals (imprecision)

^2^ Small number of events (imprecision)

^3^ High percent of patients were lost to follow-up in Elgergawy, 2019 (assumed risk of bia)

^4^ Possibility of publication bias

**Fig. 3:**
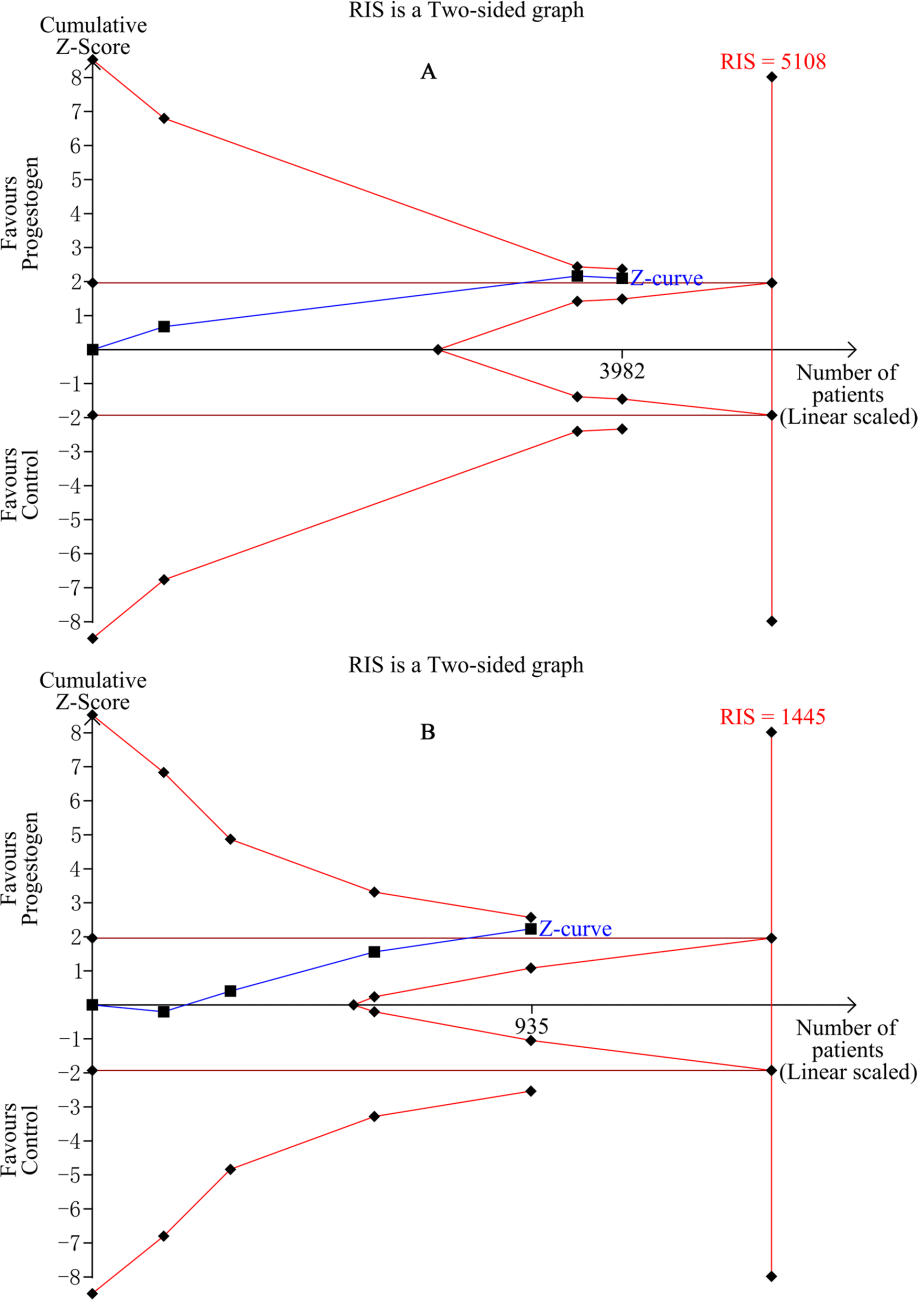
Trial sequential analysis for (**A**) preeclampsia and (**B**) low birth weight. RIS; required information size

Leave-one-out meta-analysis revealed that the outcomes of the analysis for PE and LBW were affected by the results of a single study (Fig. S7). Trial sequential analysis revealed that the sample size that was achieved to support the hypothesis of decreased risk of PE among women that received progestogen was not enough to exclude the possibility of type 1 and type 2 errors (a-spending adjusted CI 0.39–1.07). the available data were not sufficient to reach definitive conclusions in the field of LBW (a-spending adjusted CI 0.29–1.11) (Fig. 3).

## Discussion

### Principal findings

The present meta-analysis showed that early progestogen supplementation before 20 weeks of pregnancy in spontaneously achieved singleton pregnancies seemed to be associated with lower risks of PE and LBW compared with unexposed pregnant women. These results were mainly attributed to the findings of the larger studies included in our analysis and were not of adequate power according to the results of the sequential analysis. The risks of GDM, placenta previa, placental abruption, PTB, SGA and PM showed no significant differences between singleton pregnancies after progestogen supplementation versus no supplementation.

### Comparison with existing literature

Three studies were involved in the meta-analysis of PE, two of which were performed by Coomarasamy A, et al. in UK and the Netherlands. Each participant in the two trials received either micronised progesterone 400 mg twice daily or placebo capsules, to be administered vaginally. There were also no significant differences in maternal age and body-mass index of the participants in the two studies. We supposed the different results of PE between them were mainly due to the large discrepancy in sample size. The required information size for PE was 5108 participants, ten times larger than the sample size in the study in 2015, thus making the result of this study less credible with wide CI. Actually, we found the incidence of PE was slightly higher in this study than that in 2019, the difference could be explained because more than half of the participants were nulliparous in study in 2015. Nulliparity had been reported to be associated with increased probability of PE [45]. The study by Chan DMK, et al. reported negative result with the lowest risk of PE. Except for the small sample size, the demographic characteristics of participants and the progesterone regimens were supposed to contribute to the different result. In their study, the mean body-mass index of participants was 22.3 kg/m^2^ and few women were obese. Previous study demonstrated that vaginal progesterone administration, but not oral dydrogesterone could result in the decrease in the spiral artery pulsatility and resistance index and systolic/diastolic ratio [46]. Therefore, the differences observed between oral dydrogesterone and vaginal progesterone in the present meta-analysis were anticipated.

Tskhay V et al. reported a significantly lower risk of PE when dydrogesterone was used in early pregnancies with high-risk factors for developing PE. The study was retrospective and did not adjust for potential confounding factors, such as age, obesity and medical history [11]. The prevalence of PE in their control group was 13.1%, which was higher than that in our review, which may be explained by the socioeconomic and demographic differences in the participants [47]. In contrast, in our review, the prevalence of PE in the control group was approximately 3%, which was lower than that reported in a previous study, probably because the included studies did not recruit pregnant women with advanced age [48]. A prospective, comparative, cross-sectional study was performed among a group of primigravidae, which concluded that dydrogesterone supplementation during the first trimester significantly reduced the incidence of hypertension in pregnancy [49]. In that study, pregnancies with high-risk factors for gestational hypertension were excluded. However, it was inappropriate to compare women achieving pregnancy after the use of assisted reproductive techniques in the study group with those women spontaneously achieving pregnancy in the control group, given the significantly increased risk of 30% for PIH in singleton pregnancies that were created with the use of assisted reproductive techniques [50]. A previous study attempted to treat PIH and PE with progesterone in the third trimester [51]. Based on the theory that the pathological process has already started in early pregnancy, it is no surprise that progesterone supplementation in the third trimester failed to improve the patients’ blood pressure values because irreversible changes had already taken place by that point [52].

The underlying mechanisms regarding the decreased risk of PE in our review remain unclear. The pathophysiology of PE is thought to be a combination of genetic predispositions and placental dysfunction [53]. A potential explanation for the decreased risk of PE after progesterone supplementation may be its effect of lowering vascular resistance in uteroplacental blood flow and of improving endometrial blood flow, which was confirmed by Ghosh et al. [54]. In another study, PE was proved to be a state of progesterone deficiency and 17-alpha-hydroxyprogesterone caproate could blunt hypertensive actions in response to placental ischemia, without further reducing pup weight and litter size, or inducing fetal malformations [55]. In further research, 17-alpha-hydroxyprogesterone caproate was proved to blunt inflammatory cytokine secretion, hypertension, and renal endothelin-1 in a pregnant rat model of PE [56]. In a recent published study, progesterone was suggested to be able to stimulate progesterone-induced blocking factor, which was associated with improved inflammation, fetal growth restriction, and blood pressure in a rat model of PE [57].

Previous studies have reported a significantly higher incidence of GDM in women with singleton gestations receiving intramuscular 17a-hydroxyprogesterone caproate for recurrent preterm birth prevention [9, 10]. In our study, we found that the risk of GDM did not increase in pregnancies receiving vaginal or oral progestogen supplementation before 20 weeks of pregnancy. First, the differences were partly resulted from the different pharmacokinetics and medication times used in the studies. Compared with intramuscular regimens, vaginal progestogen supplementation consistently allowed for rapid progesterone absorption and achieved higher endometrial tissue concentrations with lower systemic exposures [58]. Second, the increase in plasma progesterone probably played a role in the induction of maternal hyperphagia and excessive gestational weight gain was reported to be associated with a greater risk of developing GDM [59–61]. In early gestation, pituitary growth hormone inhibits adipogenesis in primary pre-adipocytes, stimulates lipolysis and inhibits lipogenesis in mature fat cells, however, its concentrations decline during early and mid-pregnancy and are no longer detected in maternal serum after 20–24 weeks of gestation [62, 63]. So, it was unlikely to promote the developing of GDM as progesterone regimens had been administered in early gestation and stopped before 20 weeks of pregnancy in our study. Third, placental growth hormone levels are relatively low in early gestation, which increase insulin sensitivity. However, with the rise of placental growth hormone levels after mid-gestation, insulin production escalates and rates of lipolysis increase. These changes reflect the development of maternal insulin resistance [62–64].

Additionally, a lower risk of LBW was observed in pregnant women receiving oral dydrogesterone before 20 weeks of pregnancy. We believe that this effect was partly related to the lower risk of PE in the participants. Early-onset preeclampsia was suggested to mainly result from early placental dysfunction which had severe adverse effects on fetal growth [65, 66]. Previous study showed that dydrogesterone could reduce resistance index of uterine artery and middle cerebral and increase fetal weight [67].

### Strengths and limitations

Our study had unique strengths. First, this review is an up-to-date evidence study and the first study to comprehensively investigate the association of early progestogen supplementation before 20 weeks of pregnancy in spontaneously achieved singleton pregnancies and pregnancy-related complications. Second, only spontaneously conceived pregnancies were included, and there was no heterogeneity observed among the studies, thus providing more precise and reliable risk estimates. Finally, all the studies included in the meta-analysis were high quality, and unpublished information was obtained from the original authors.

Our study has several limitations. First, the definitions of the outcomes were not reported or were unclear in several studies, making it difficult to adapt to uniform standards, which may have caused our results to be underestimated or overestimated. Second, the types, doses and routes of progestogen differed among the included studies, thus resulting in clinicians being uncertain as to which treatment to choose. Third, some participants conceived after the use of assisted reproductive technology (less than 2%), and 10 twin pregnancies were included in the meta-analysis; however, such a small fraction of patients were unlikely to affect the main results. Fourth, most of the studies did not report on the use of aspirin. However, if aspirin is used in both groups, then more women in the progestogen groups are expected to be prevented from developing PE than the control groups, which will pull the risk estimate towards the null. Fifth, our review does not address other populations of potential interest, including pregnant women with chronic hypertension and those with PE in the previous pregnancy. Finally, although we searched three electronic databases and ClinicalTrials.gov, only nine studies were included in the present review and conference abstracts, crossover trials were excluded. Thus, evidence certainty was downgraded due to concern about publication bias. Many of these complications were not reported in past studies, or only included as secondary end-points in the included studies. We could not rule out the possibility that these studies didn’t report these outcomes because no statistically significant differences were observed. Our results might have been affected by this issue.

### Clinical implications

The findings of our meta-analysis indicate that progestogen supplementation before 20 weeks of pregnancy in spontaneously achieved singleton pregnancies may reduce the risk of both PE and LBW. However, current available data is not sufficient to reach definitive conclusions and the results should be interpreted with caution. Given the uncertain benefit and relatively low incidence of PE in these women, it is unlikely to promote the application of progestogen supplementation in clinical practice. However, women should be informed of this information before the application of progestogen therapy before 20 weeks of pregnancy. Future studies are needed to accumulate the evidence and to explore the ideal group of women who will have the highest likelihood of benefitting from progestogen supplementation. Our work showed that early progestogen supplementation before 20 weeks of pregnancy in spontaneously achieved singleton pregnancies appeared to yield at least equivalent or better maternal and perinatal outcomes; therefore, physicians do not need to enhance their vigilance during the antenatal management of these women.

## Conclusion

Use of vaginal micronized progesterone (Utrogestan) in spontaneously achieved singleton pregnancies with threatened miscarriage before 20 weeks of pregnancy may reduce the risk of PE in later gestational weeks. Among spontaneously achieved singleton pregnancies with threatened miscarriage or a history of recurrent miscarriage, use of oral dydrogesterone before 20 weeks of pregnancy may result in a lower risk of LBW in later gestational weeks, however, available data was not adequately powered and the total sample size of enrolled women did not reach the required information size. Future studies are needed to accumulate the evidence and to explore the ideal group of women who will have the highest likelihood of benefitting from progestogen supplementation in early gestational weeks.

## Supplementary Information

**Additional file 1: Figure S1.** Risk of bias assessment for included studies. **Figure S2.** Funnel plots for the outcomes. **Figure S3.** Subgroup analyses of preeclampsia. **Figure S4.** Forest plot diagram of secondary maternal outcomes. **Figure S5.** Forest plot diagrams of perinatal outcomes. **Figure S6.** Subgroup analyses of low birth weight. **Figure S7.** Leave one out meta-analysis for (A) preeclampsia and (B) low birth weight.

**Additional file 2: Table S1.** Characteristics of included studies and reasons for exclusion in study selection.

**Additional file 3: Appendix S1.** Detailed search strategy.

## Data Availability

The current study was based on the results of relevant published studies.

## Abbreviations

ACOG: American College of Obstetricians and Gynecologists
GDM: Gestational diabetes mellitus
PE: Preeclampsia
RCTs: Randomized controlled trials
LBW: Low birth weight
VLBW: Very low birth weight
PTB: Preterm birth
SGA: Small for gestational age
LGA: Large for gestational age
ORs: Odds ratios
CIs: Confidence intervals
TSA: Trial sequential analysis
