# Supplementary figures and images for: Pregnancy-related complications and perinatal outcomes following progesterone supplementation before 20 weeks of pregnancy in spontaneously achieved singleton pregnancies: a systematic review and meta-analysis
Source: Reprod Biol Endocrinol. 2021 Nov 4;19:165. doi: 10.1186/s12958-021-00846-6 (PMC8567546; doi:10.1186/s12958-021-00846-6)

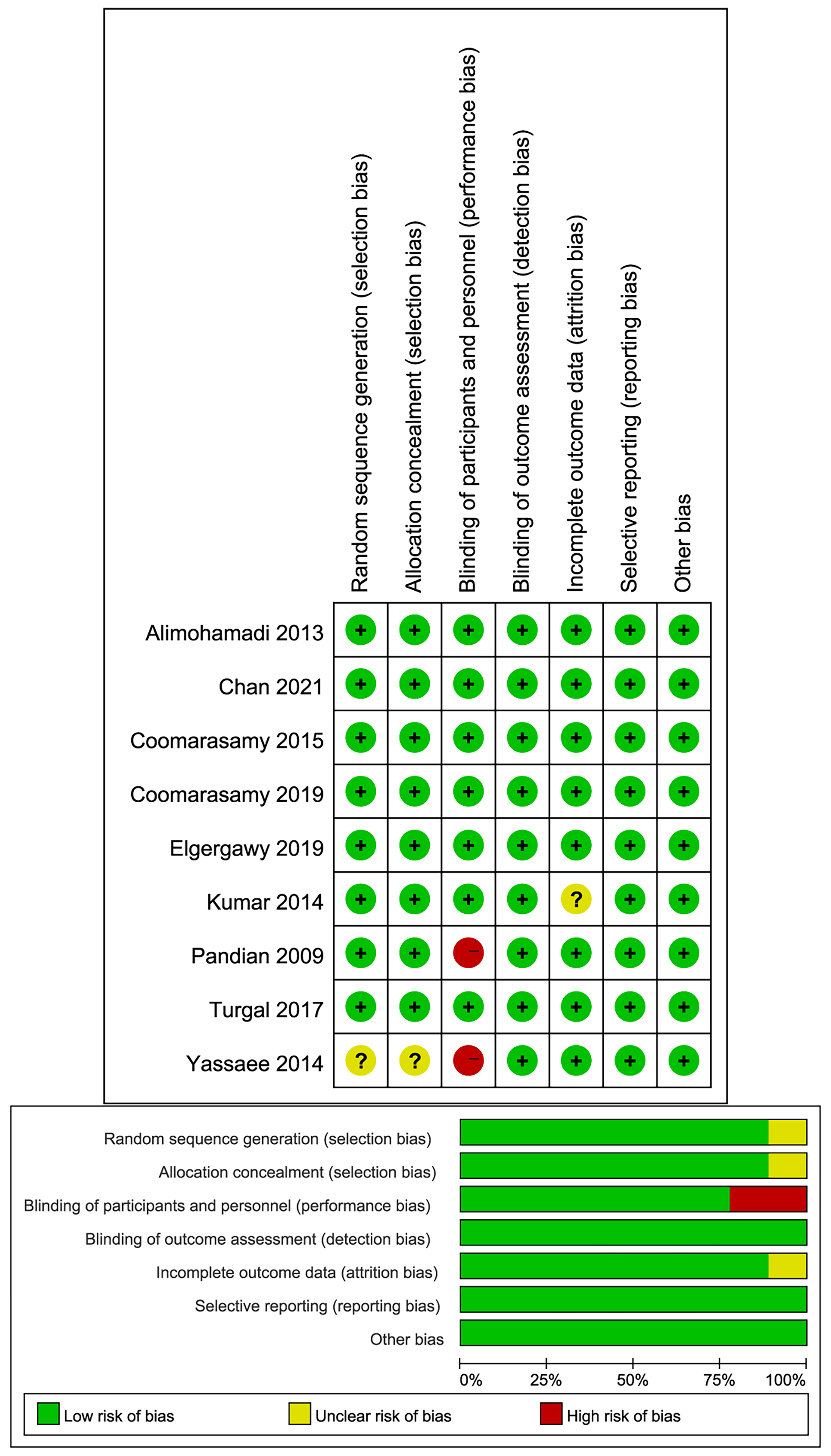

Supplement: Supplementary file 1 — Additional file 1: Figure S1. Risk of bias assessment for included studies. Figure S2. Funnel plots for the outcomes. Figure S3. Subgroup analyses of preeclampsia. Figure S4. Forest plot diagram of secondary maternal outcomes. Figure S5. Forest plot diagrams of perinatal outcomes. Figure S6. Subgroup analyses of low birth weight. Figure S7. Leave one out meta-analysis for (A) preeclampsia and (B) low birth weight. [file 12958_2021_846_MOESM1_ESM.zip › Figure S1. Risk of bias assessment for included studies.tif]

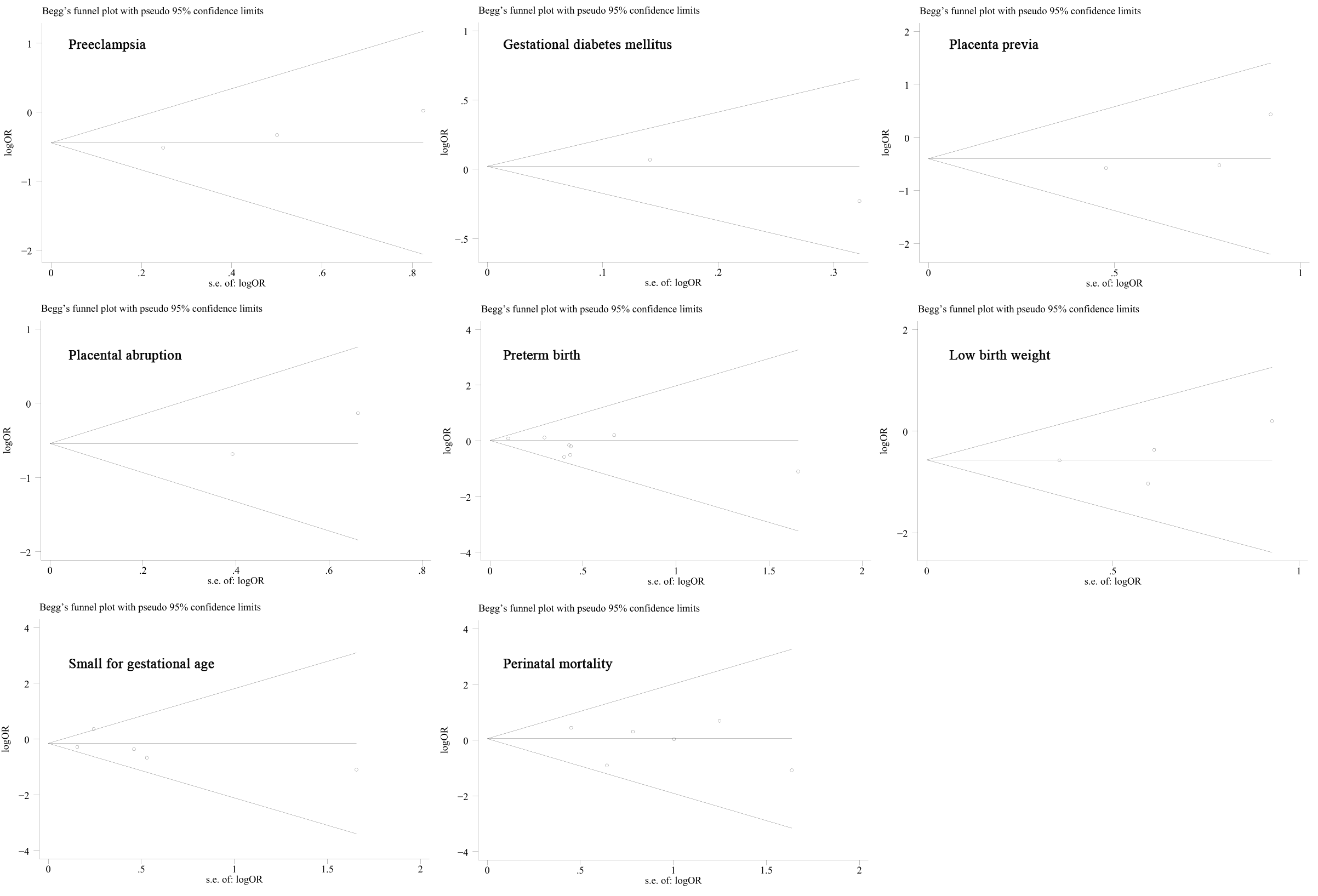

Supplement: Supplementary file 1 — Additional file 1: Figure S1. Risk of bias assessment for included studies. Figure S2. Funnel plots for the outcomes. Figure S3. Subgroup analyses of preeclampsia. Figure S4. Forest plot diagram of secondary maternal outcomes. Figure S5. Forest plot diagrams of perinatal outcomes. Figure S6. Subgroup analyses of low birth weight. Figure S7. Leave one out meta-analysis for (A) preeclampsia and (B) low birth weight. [file 12958_2021_846_MOESM1_ESM.zip › Figure S2. Funnel plots for the outcomes.tif]

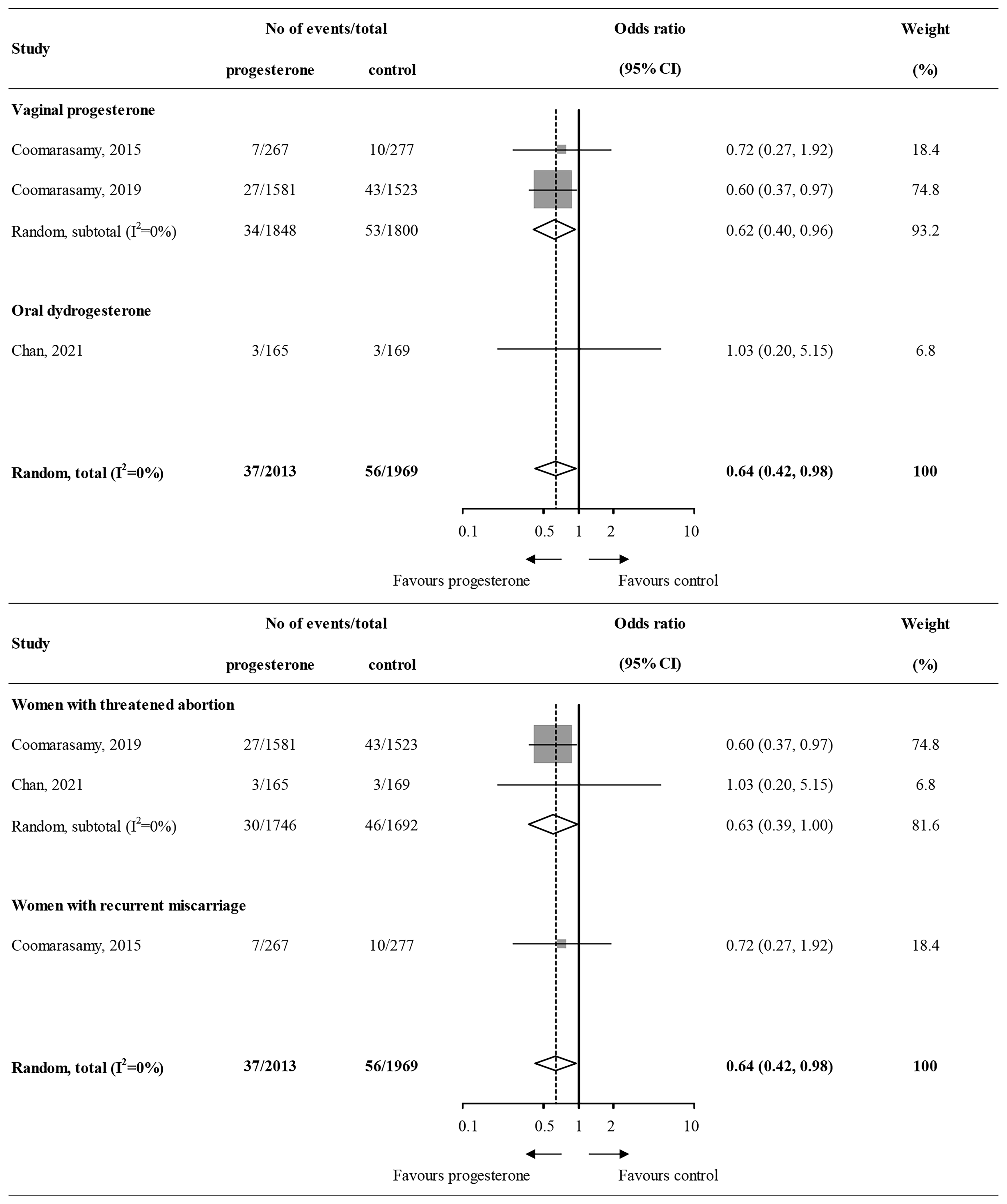

Supplement: Supplementary file 1 — Additional file 1: Figure S1. Risk of bias assessment for included studies. Figure S2. Funnel plots for the outcomes. Figure S3. Subgroup analyses of preeclampsia. Figure S4. Forest plot diagram of secondary maternal outcomes. Figure S5. Forest plot diagrams of perinatal outcomes. Figure S6. Subgroup analyses of low birth weight. Figure S7. Leave one out meta-analysis for (A) preeclampsia and (B) low birth weight. [file 12958_2021_846_MOESM1_ESM.zip › Figure S3.tif]

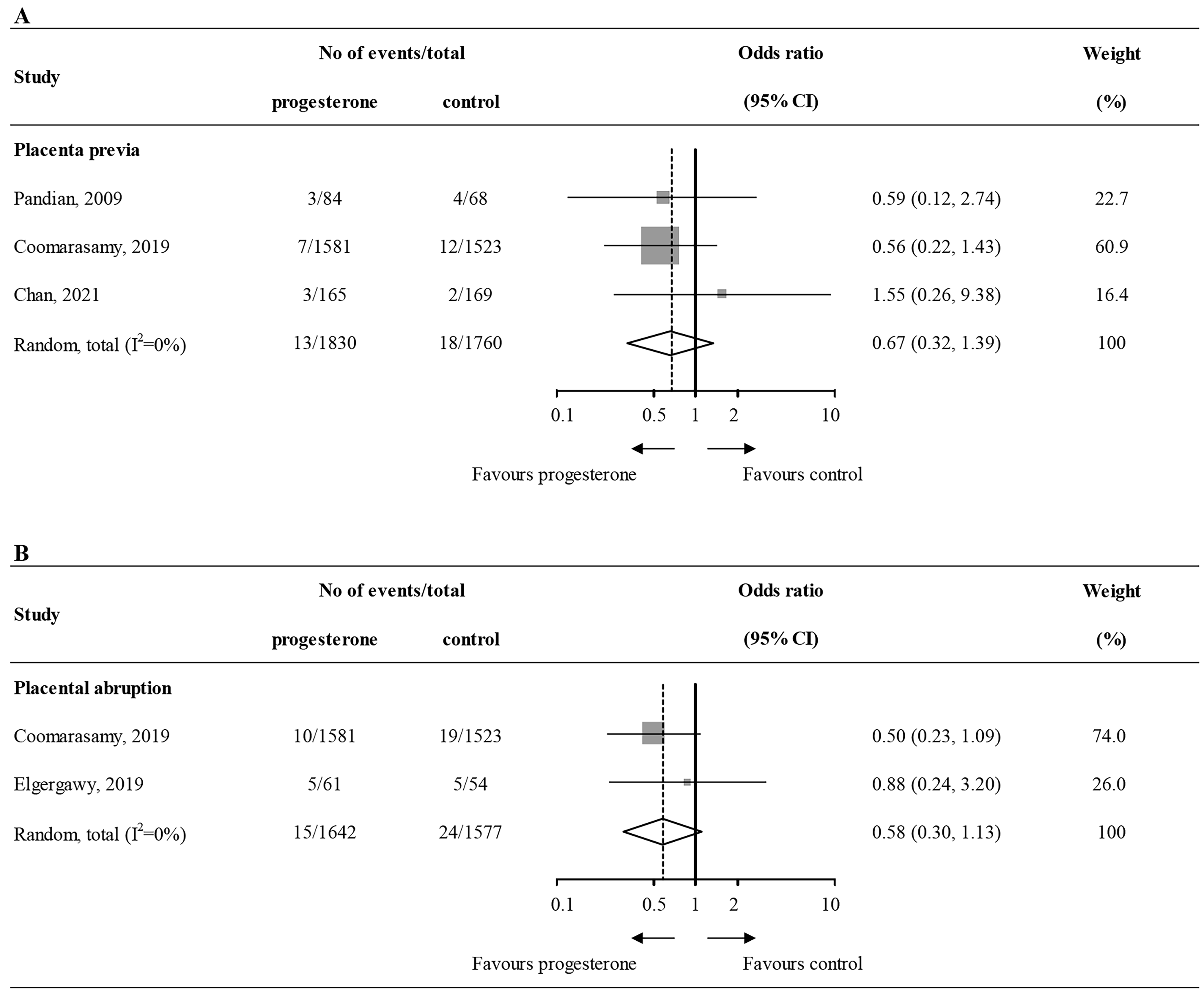

Supplement: Supplementary file 1 — Additional file 1: Figure S1. Risk of bias assessment for included studies. Figure S2. Funnel plots for the outcomes. Figure S3. Subgroup analyses of preeclampsia. Figure S4. Forest plot diagram of secondary maternal outcomes. Figure S5. Forest plot diagrams of perinatal outcomes. Figure S6. Subgroup analyses of low birth weight. Figure S7. Leave one out meta-analysis for (A) preeclampsia and (B) low birth weight. [file 12958_2021_846_MOESM1_ESM.zip › Figure S4.tif]

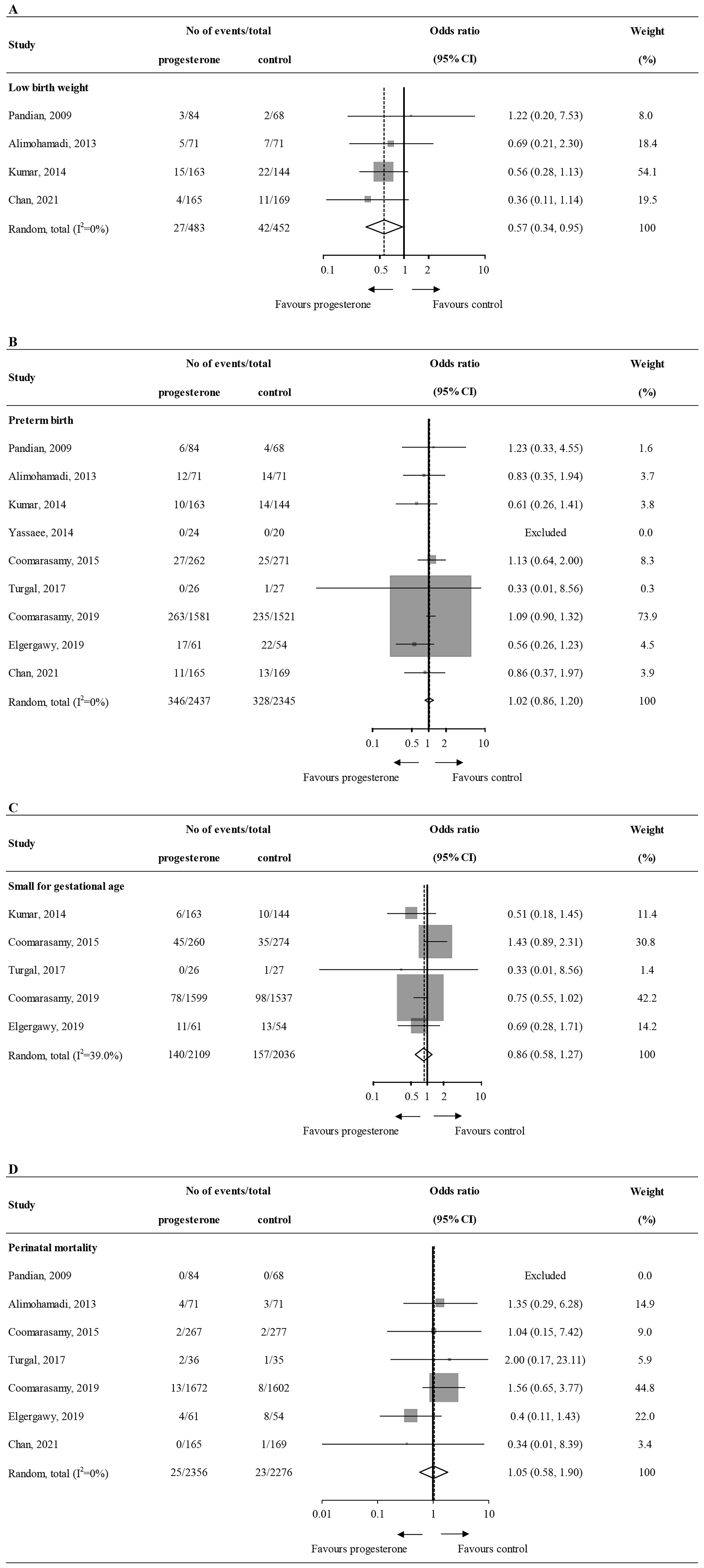

Supplement: Supplementary file 1 — Additional file 1: Figure S1. Risk of bias assessment for included studies. Figure S2. Funnel plots for the outcomes. Figure S3. Subgroup analyses of preeclampsia. Figure S4. Forest plot diagram of secondary maternal outcomes. Figure S5. Forest plot diagrams of perinatal outcomes. Figure S6. Subgroup analyses of low birth weight. Figure S7. Leave one out meta-analysis for (A) preeclampsia and (B) low birth weight. [file 12958_2021_846_MOESM1_ESM.zip › Figure S5.tif]

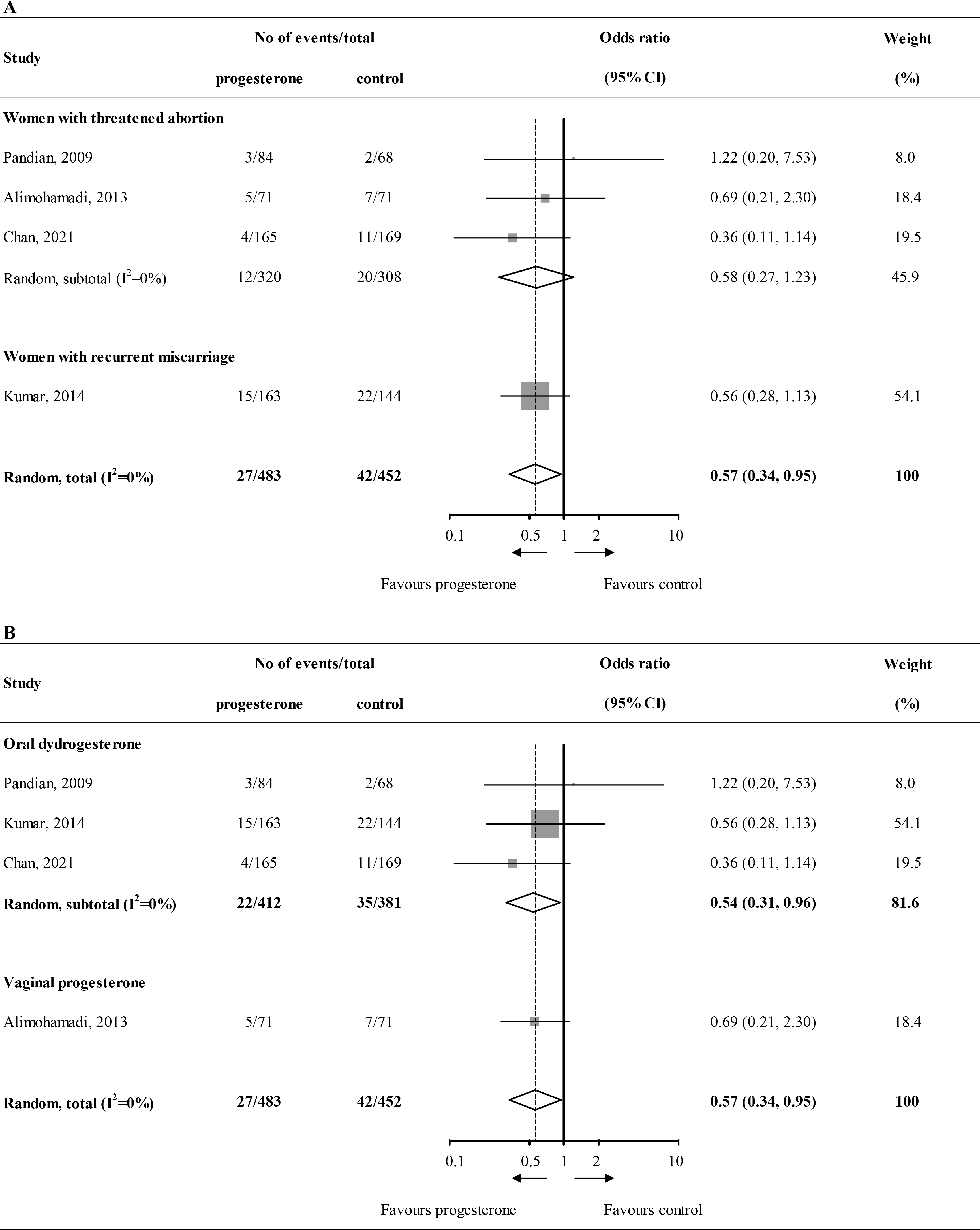

Supplement: Supplementary file 1 — Additional file 1: Figure S1. Risk of bias assessment for included studies. Figure S2. Funnel plots for the outcomes. Figure S3. Subgroup analyses of preeclampsia. Figure S4. Forest plot diagram of secondary maternal outcomes. Figure S5. Forest plot diagrams of perinatal outcomes. Figure S6. Subgroup analyses of low birth weight. Figure S7. Leave one out meta-analysis for (A) preeclampsia and (B) low birth weight. [file 12958_2021_846_MOESM1_ESM.zip › Figure S6.tif]

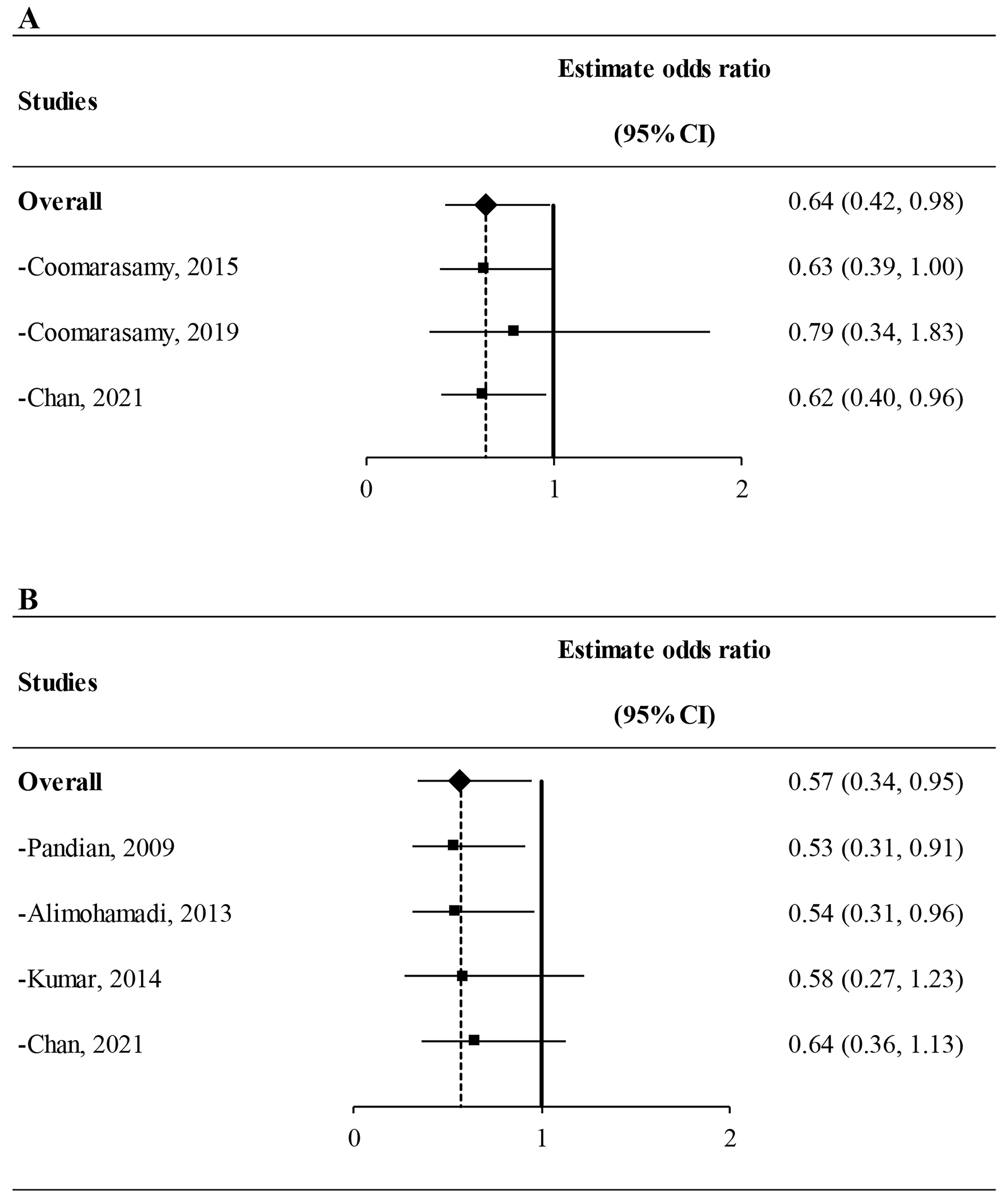

Supplement: Supplementary file 1 — Additional file 1: Figure S1. Risk of bias assessment for included studies. Figure S2. Funnel plots for the outcomes. Figure S3. Subgroup analyses of preeclampsia. Figure S4. Forest plot diagram of secondary maternal outcomes. Figure S5. Forest plot diagrams of perinatal outcomes. Figure S6. Subgroup analyses of low birth weight. Figure S7. Leave one out meta-analysis for (A) preeclampsia and (B) low birth weight. [file 12958_2021_846_MOESM1_ESM.zip › Figure S7. Leave one out meta-analysis.tif]
