# Supplementary material for: Pregnancy-related complications and perinatal outcomes following progesterone supplementation before 20 weeks of pregnancy in spontaneously achieved singleton pregnancies: a systematic review and meta-analysis
Source: Reprod Biol Endocrinol. 2021 Nov 4;19:165. doi: 10.1186/s12958-021-00846-6 (PMC8567546; doi:10.1186/s12958-021-00846-6)
Supplement: Supplementary file 3 — Additional file 3: Appendix S1. Detailed search strategy. [file 12958_2021_846_MOESM3_ESM.docx]

**Appendix S1. Detailed search strategy**

Search strategy for Ovid MEDLINE, the Cochrane Library and Embase

EBM Reviews - Cochrane Central Register of Controlled Trials <February 2021>

EBM Reviews - Cochrane Database of Systematic Reviews <2005 to March 31, 2021>

EBM Reviews - Cochrane Clinical Answers <March 2021>

EBM Reviews - Cochrane Methodology Register <3rd Quarter 2012>

EBM Reviews - Database of Abstracts of Reviews of Effects <1st Quarter 2016>

Embase <1974 to 2021 April 01>

Ovid Emcare <1995 to 2021 Week 12>

Ovid MEDLINE(R) and Epub Ahead of Print, In-Process, In-Data-Review & Other Non-Indexed Citations, Daily and Versions(R) <1946 to April 01, 2021>

1 exp progesterone/ 175026

2 algestone/ or algestone acetophenide/ or 20-alpha-dihydroprogesterone/ or hydroxyprogesterones/ or 17-alpha-hydroxyprogesterone/ or medroxyprogesterone/ or medroxyprogesterone acetate/ or (progesterone or ("17" adj3 hydroxyprogesterone)).mp. 323573

3 progestin.tw. 23352

4 progestogen.tw. 10206

5 dydrogesterone.tw. 1767

6 utrogest.tw. 63

7 prontogest.tw. 166

8 1 or 2 or 3 or 4 or 5 or 6 or 7 343411

9 exp Hypertension, Pregnancy-Induced/ or exp Pre-Eclampsia/ 123781

10 (Hypertens* and Pregnan*).mp. [mp=ti, ot, ab, sh, hw, kw, tx, ct, tn, dm, mf, dv, fx, dq, nm, kf, ox, px, rx, an, ui, sy] 108648

11 (Preeclamp* or Pre-eclamp* or eclamp*).tw. 106964

12 exp Diabetes, Gestational/ 63520

13 gestational diabetes.mp. 52859

14 pregnancy induced diabetes.mp. 41

15 pregnancy-induced diabetes.mp. 41

16 GDM.mp. 26745

17 exp Placenta previa/ 11098

18 Placenta abruption.tw. 328

19 exp Premature Birth/ 164770

20 Preterm.tw. 236829

21 exp Postpartum Hemorrhage/ 27575

22 (Hemorrhage, Postpartum or Immediate Postpartum Hemorrhage or Hemorrhage, Immediate Postpartum or Postpartum Hemorrhage, Immediate or post-partum haemorrhage or postpartum haemorrhage or post-partum hemorrhage).mp. 11173

23 ((neonatal or perinatal or fetal or birth$ or deliver$) adj2 outcome$).ti,ab. 116266

24 exp Stillbirth/ 30091

25 (stillborn or stillbirth).ti,ab. 32061

26 ((f?etal or intrauterine or intra-uterine) adj2 (growth or death$ or loss$)).ti,ab. 116532

27 IUGR.ti,ab. 17460

28 (small adj2 gestational age).ti,ab. 31260

29 (large adj2 gestational age).ti,ab. 7836

30 exp Perinatal mortality/ 38225

31 Pregnancy complications.tw. 18697

32 Obstetric complications.tw. 9785

33 Pregnancy outcomes.tw. 47046

34 Birth outcomes.tw. 15892

35 Obstetric outcomes.tw. 5191

36 adverse outcomes.tw. 76441

37 pregnanc*.ti,ab. 1159319

38 9 or 10 or 11 or 12 or 13 or 14 or 15 or 16 or 17 or 18 or 19 or 20 or 21 or 22 or 23 or 24 or 25 or 26 or 27 or 28 or 29 or 30 or 31 or 32 or 33 or 34 or 35 or 36 or 37 1664406

39 8 and 38 58291

40 exp animals/ not humans.sh. 37070731

41 39 not 40 18761

42 randomized controlled trial.pt. 1039802

43 controlled clinical trial.pt. 186099

44 randomized.ab. 2081315

45 randomised.ab. 424525

46 placebo.tw. 948062

47 clinical trials as topic.sh. 228572

48 randomly.ab. 1243279

49 trial.ti. 1014753

50 42 or 43 or 44 or 45 or 46 or 47 or 48 or 49 4653966

51 41 and 50 4299

Search strategy for ClinicalTrials.gov

Condition or disease: pregnancy

Other terms: progesterone

Search Results: 291
